# Cribriform Morular Thyroid Carcinoma – Ultimobranchial Pouch-Related? Deep Molecular Insights of a Unique Case

**DOI:** 10.1007/s12022-023-09775-z

**Published:** 2023-05-30

**Authors:** Matthias S. Dettmer, Sandra Hürlimann, Lukas Scheuble, Erik Vassella, Aurel Perren, Corinna Wicke

**Affiliations:** 1 Institute of Pathology, University of Bern, Bern, Switzerland; 2 Institute of Surgical Pathology, LUKS, Lucerne, Switzerland; 3 Thyroid Center LUKS, Lucerne, Switzerland; 4 Institute of Pathology, Klinikum Stuttgart, Kriegsbergstrasse 60, 70174 Stuttgart, Germany

**Keywords:** Thyroid carcinoma, FAP, Clonal evolution, Cribriform morular, Squamous cell component, NGS

## Abstract

A 44-year-old female patient with a familial adenomatous polyposis (FAP) was diagnosed with a cribriform morular thyroid carcinoma (CMTC). We observed within the very necrotic tumor a small but distinct poorly differentiated carcinomatous component. As expected, next generation sequencing of both components revealed a homozygous *APC* mutation and in addition, a *TERT* promoter mutation. A *TP53* mutation was found exclusively in the CMTC part, while the poorly differentiated component showed a clonal evolution, harboring an activating *PIK3CA* mutation and copy number gains of *BRCA2*, *FGF23*, *FGFR1*, and *PIK3CB*—alterations which are typically seen in squamous cell carcinoma. The mutational burden in both components was low, and there was no evidence for microsatellite instability. No mutations involving the mitogen-activated protein kinase (MAPK) pathway, typically seen in papillary thyroid carcinomas, were detected. Immunohistochemically, all tumor parts were negative for thyroglobulin, providing further evidence that this entity does not belong to the follicular epithelial cell-derived thyroid carcinoma group. CD5 was negative in the poorly differentiated component, making a relation to intrathyroidal thymic carcinoma rather unlikely. However, since this marker was seen in the morules, a loss in the poorly differentiated component and a relation to the ultimobranchial body cannot be excluded either. After total thyroidectomy and radioiodine ablation, the patient was disease-free with no residual tumor burden on 2-year follow-up.

## Introduction

Cribriform morular thyroid carcinoma (CMTC) is frequently associated with familial adenomatous polyposis (FAP) and is characterized by a strong female predominance [1]. It has an excellent prognosis and a very distinctive histology with multifocal morules within the neoplasm. Because of this distinctive morphology, it serves as an indicator lesion for FAP which is present in about 40% of CMTC patients and which is much more likely to determine the patient’s fate with multiple colonic adenomas and a very high lifetime risk of progression to invasive colorectal cancer.

This report is, to our knowledge, the first case of a CMTC with morphological and molecular clonal evolution into a poorly differentiated carcinoma with squamoid morphology without invasion into perilesional tissue, and we provide next generation sequencing data of both tumor components with new molecular insights into tumor progression of this rare TC (thyroid carcinoma) variant.

## Patient’s Clinical History

A 44-year-old female patient was referred to our thyroid center with a painful left cervical mass. She has a family history of FAP which is genetically confirmed. Nineteen years earlier, she underwent a prophylactic proctocolectomy, which showed no malignancy. There was no positive family history for thyroid or parathyroid disease. Due to hypothyroidism, the patient has been on hormonal substitution therapy with levothyroxine for the past 6 years with stable hormonal function. B-symptoms were denied. On inspection, she had a grade 2 left-sided goiter. Physical examination showed a palpable thyroid nodule which was not tender or indurated without adjacent cervical lymphadenopathy. The patient was euthyroid; serum calcium and calcitonin were in the normal range, and thyroid antibodies were negative. Sonographically, the left thyroid lobe was almost completely filled by a hypoechogenic nodule (EU-TIRADS 4). The right lobe of the thyroid gland was free of nodules. No evidence of lymphadenopathy could be found in the cervicocentral or cervicolateral compartments. Fine-needle aspiration revealed foam cells and blood. No epithelia could be detected. The findings were consistent with cystic fluid (Bethesda category I). Initially, a left hemithyroidectomy was performed without complications.

**Fig. 1:**
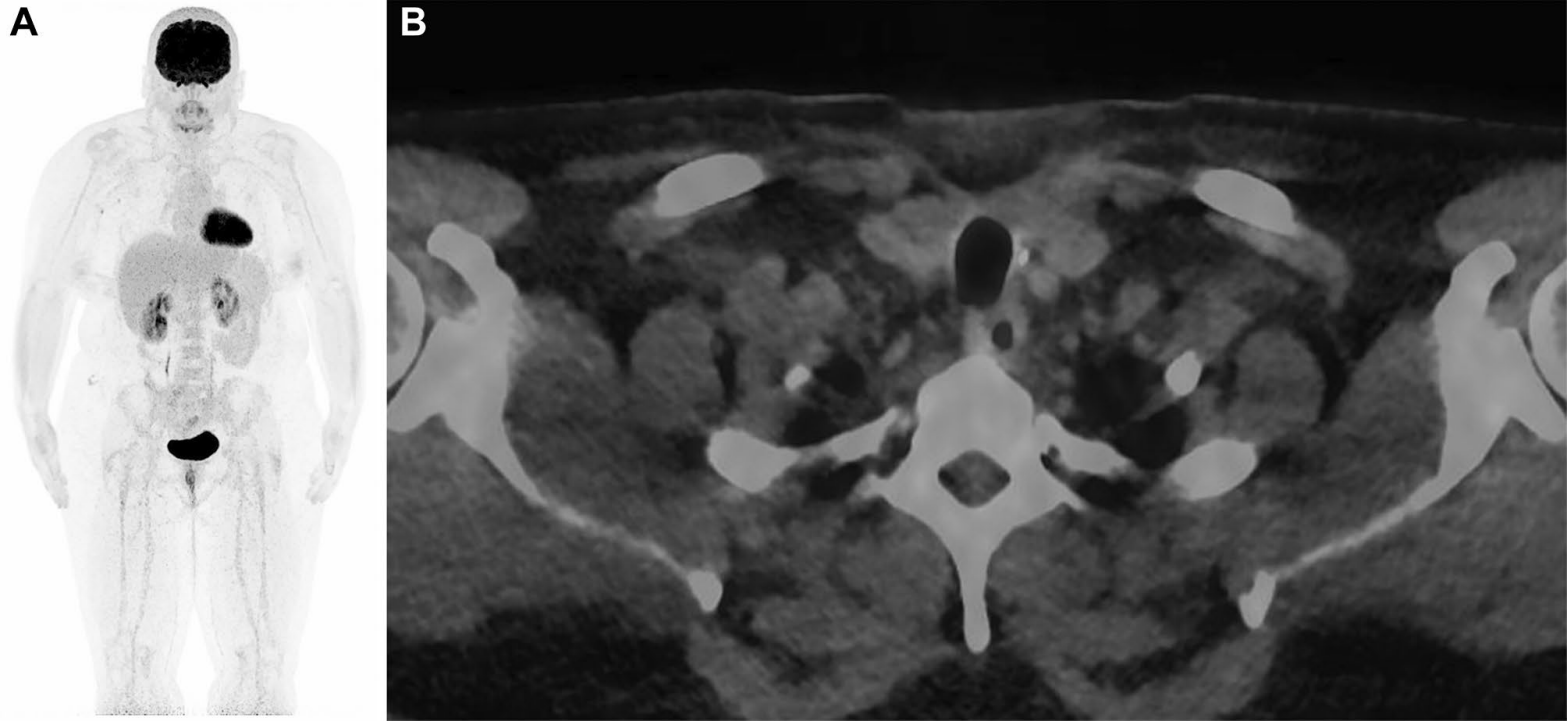
**a** and **b** FDG-PET/CT imaging 6 months postoperatively showing no sign of systemic or locoregional disease

After obtaining the definite histological results and collecting international expert opinions, the case was extensively discussed at our interdisciplinary tumor board for endocrine malignancies. The recommendation was made for staging by whole-body FDG-PET-CT and subsequent completion thyroidectomy as well as adjuvant radioiodine therapy which was performed without complications. Whole-body FDG-PET-CT showed no local or distant metastases. Postoperatively, the patient had normal serum calcium and parathyroid hormone values and a normal phoniatrical evaluation. No further malignant tissue was found in the final histology. After completion of wound healing, adjuvant radioiodine therapy with 1958 MBq iodine 131 under exogenous stimulation was performed 4 weeks postoperatively. Thyroid hormone substitution was continued and adjusted (TSH goal 0.5–2.0 mU/L). At the first follow-up, 6 months postoperatively, clinical examination, sonography, posttreatment whole-body radioiodine scan, and partial body PET/CT with 274 MBq 18F-FDG showed no evidence of local recurrence in the thyroid bed, suspicious lymph nodes, or distant metastases. The patient had a TSH-stimulated thyroglobulin of < 1 ng/mL. The last follow-up, 2 years postoperatively, including physical examination, non-stimulated serum thyroglobulin, and imaging showed a persisting excellent response to initial therapy (Fig. 1a and b).

## Materials and Methods

**Fig. 2:**
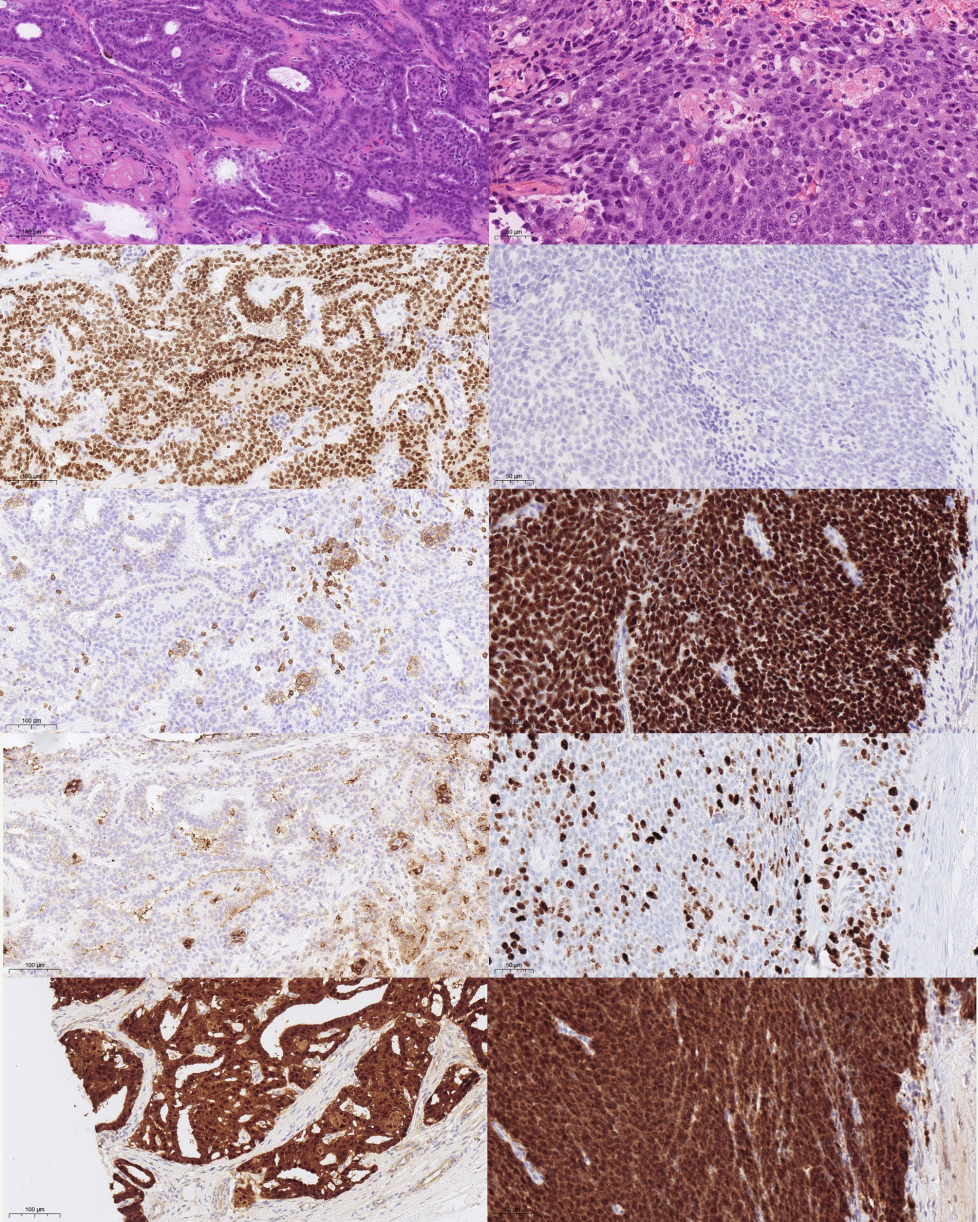
Left side, well-differentiated CMT component, 20 × magnification, right side poorly differentiated areas, 30 × magnification. First row HE. Second row TTF1, note negativity in the morules. Third row left: CD 5, with positivity in morules, negativity in the poorly differentiated areas (not shown). Third row right: p40, strong nuclear positivity in poorly differentiated areas. Forth row left: CD10 partial positivity in morules. Forth row right: increased Ki-67 in poorly differentiated areas. Fifth row: strong cytoplasmic and nuclear b-catenin staining in both areas

Reference histology showed a 3-cm largely necrotic tumorous mass. In the vital parts, the classic hallmarks of CMTC were seen including complex architecture with cribriform and squamoid morulae without colloid formation [1, 2]. Immunohistochemistry showed TTF1 (Clone SPT24, Novocastra, cat-nr.:NCL-L-TTF1) and estrogen receptor positivity and diffuse nuclear accumulation of beta-catenin and a negativity for PAX-8 (polyclonal, Proteintech, cat-nr.: 10,336–1-AP) and thyroglobulin, the squamoid morules were positive for CD10 and negative for TTF1, estrogen receptor, PAX-8 and thyroglobulin (Fig. 2, Table 1). More important, there was a small but very unique second tumor component of a poorly differentiated carcinoma. No evidence of lymphatic or vascular invasion was found. Both tumor parts were sequenced using Illuminas true sight oncology 500 (TSO500) panel on DNA and RNA level. There was a homozygous *APC* mutation in both tumor parts with a high allelic frequency, consistent with the diagnosed FAP of the patient. A *TERT* promoter mutation was also identified in both tumor areas. A *TP53* mutation was found exclusively in the CMTC part. The poorly differentiated carcinoma only harbored an activating *PIK3CA* mutation and copy number gains of *BRCA2*, *FGF23*, *FGFR1*, and *PIK3CB* (Table 2). The mutational burden in both components was 3.9 mutations per megabase, and there was no evidence for microsatellite instability (instability in 2 of 125 microsatellites). Of note, a metastasis of a squamous cell carcinoma primary into the CMTC was ruled out clinically and on molecular grounds since it shared some mutations with the CMTC.

**Table 1:** Immunohistochemical results in the different tumor components

	CMTC	CMTC-morules	Dedifferentiated component
TTF1	Pos	Neg	Neg
CD5	Neg	Pos	Neg
p40	Neg	Neg	Pos
CD10	Neg	Pos	Neg
KI67	Low	Low	Increased
PAX8	Neg	Neg	Neg
CK5	Neg	Neg	Neg
Thyroglobulin	Neg	Neg	Neg
Estrogen receptor	Neg	Neg	Neg
PanCK	Pos	Pos	Pos

**Table 2:** Molecular results in the different tumor components

	CMTC	Dedifferentiated component
*APC* E1257R (c.3768dup)	+ (84.8%)	+ (92.3%)
TERT 228C > T	+ (29.4%)	+ (45.8%)
*TP53* C275Wfs (c.824dup)	+ (9.9%)	-
*PIK3CA* E110del (c.328_330del)	-	+ (26.7%)
*PIK3CB* CNV	-	+ (3 copies)
*FGFR1* CNV	-	+ (3 copies)
*FGF23* CNV	-	+ (3 copies)
*BRCA2* CNV	-	+ (3 copies)

In brackets, allelic frequency of the mutation

## Discussion

Until recently, CMTC was thought to be a part of the spectrum of papillary thyroid carcinoma (PTC), and this is the reason why old papers list CMTC together with PTC [3]. However, new research data showed convincing evidence that CMTC is a distinct entity [2, 4].

CMTC is very rare—it occurs in only about 0.5% of all TC [5, 6]. This variant is known to occur almost exclusively in females, and it is known for a very favorable outcome [3, 4, 7, 8]. A large series of 30 CMTCs out of 17,062 PTC cases found only one patient with a local recurrence in the remaining thyroid, while all other patients were tumor-free in their long-term follow-up. Therefore, the authors did not recommend lymph node dissection in these patients [8]. It is nevertheless important to recognize this entity for pathologists and clinicians alike since it may serve as an indicator lesion for FAP, which was also present in our patient [8].

Patients with FAP frequently harbor germline mutations in *APC*, which causes activation of the Wnt signaling pathway. In the case of sporadic CMTC, this pathway can also be activated by somatic *APC* mutations or mutations in *CTNNB1*. As a consequence, β-catenin shows immunohistochemically an aberrant nuclear expression, especially in the squamoid morules, a fact that is used as an immunohistochemical diagnostic hallmark for this tumor [1, 2, 8].

The cell of origin for CMTC is still unknown. This tumor is never immunohistochemically positive for markers expressed by all other tumors derived from the follicular epithelial cell like thyroglobulin or PAX8. The immunohistochemical profile reported in the largest series of CMTC with different expression of various markers in the cribriform component and the morules are also recapitulated by the tumor presented here—at least in the well-differentiated component and the morules.

Typical PTC frequently harbors activating mutations of genes in the mitogen-activated protein kinase (MAPK) pathway, which are the main driver of tumorigenesis. These include *BRAF* and *RAS* mutations or *CCDC6*::*RET* and *NCOA4*::*RET* fusions [9]. In contrast, no *BRAF* mutation was found in a series of 17 CMTC, and only one *KRAS* mutation was reported in 7 cases [10]. The present case also had none of these mutations, providing further evidence that CMTC is a distinct neoplasm.

The differential diagnosis for the poorly differentiated component was a squamous cell carcinoma (SCC), a carcinoma arising in morules with squamous metaplasia or an intrathyroidal thymic carcinoma. Morphologically and immunohistochemically, it would best fit the diagnosis of poorly differentiated squamous cell carcinoma (SCC) because of strong nuclear positivity of p40, a marker considered very sensitive for differentiating SCC [11]. Staining for CD5 and CK5 was negative, with the latter marker being negative in approximately 5% of SCC, arguing against the diagnosis of SCC [12]. This component was seen within the CMTC without extension or invasion into the surrounding thyroid tissue and was therefore considered non-invasive, analogous to the diagnostic criteria for higher-grade TC. The possibility that the poorly differentiated component originated in ultimobranchial pouch-related cellular components, as discussed by Boyraz et al., and subsequently lost the typical morular immunophenotype, cannot be excluded and may be supported by an absence of CK5 expression [4].

The *PI3K*/*Akt* pathway plays an important role in the tumorigenesis of thyroid cancer [13], and two of the genes we identified in the poorly differentiated carcinoma component, *PIK3CA* and *PIK3CB*, belong to this pathway. However, *PIK3CA* mutations are rare in PTC, accounting for only 2% of cases, and to our knowledge have not been observed in CMTC. *FGFR1* copy number increases were also not detected in PTC or other thyroid carcinomas [14, 15].

In contrast, *PIK3CA* mutations and copy number gains of components of *FGFR* signaling pathway have been described as oncogenic drivers of squamous cell carcinoma of different origin including lung, head and neck, and stomach [16]. Therefore, we can speculate that the acquisition of these changes may have triggered the squamous morphology.

The molecular alterations just mentioned must have arisen in a subclone of CMTC prior to the acquisition of the *TP53* mutation, thereby progressing into a poorly differentiated carcinoma. *TP53* mutations are usually associated with an aggressive nature of the tumor being most common in anaplastic thyroid carcinoma [17, 18]. However, the *TP53* mutation was identified in the well-differentiated tumor area of CMTC only. The reason for this is unknown but may be explained by the different genetic background of both components. To date, no *TP53* mutations have been reported in CMTC [19].

The CMTC and the poorly differentiated cell component share a common mutation in the *TERT* promoter, indicating that they are derived from the same cell of origin. Interestingly, *TERT* promoter mutations were observed in a subset (22%) of intrathyroidal thymic carcinomas [20]. These mutations are strongly associated with an adverse clinical outcome in thyroid carcinomas [21, 22]. One publication reported a *TERT* promoter mutation in a patient with CMTC with local and distant metastases [10], consistent with the adverse outcome associated with *TERT* promoter mutations [19]. Furthermore, progression into a poorly differentiated TC with an adverse outcome including local and distant metastases has been very rarely reported in CMTC [23]. The aggressive morphology of the carcinoma component on the one hand and the presence of the *TERT* mutation on the other hand indicate a rather unfavorable prognosis of the patient.

Moreover, the new 2022 WHO classification of endocrine organs lists primary squamous cell carcinoma in the thyroid gland as part of anaplastic thyroid carcinoma [1]. However, the poorly differentiated component was encapsulated, and applying general pathologic diagnostic rules, the diagnosis of this tumor part does not qualify as poorly differentiated thyroid carcinoma according to the Turin classification or as anaplastic thyroid carcinoma, because of the absence of unequivocal invasive features [24–26]. Nevertheless, this reflects a cytomorphologically aggressive part, also underscored by the high proliferation index. A similar situation is described in thyroid tumors of follicular cell origin harboring areas of poorly differentiated thyroid carcinoma without invasion, and these tumors did not recur over a 10-year period [27]. In line with this, our patient had no evidence of local or distant metastatic spread after a 2-year follow-up. Thus, the clinical significance of genetic alterations of the CMTC case described here remains to be seen.

## Conclusion

We report a rare case of a CMTC with a poorly differentiated tumor component with squamous cell morphology. Next-generation sequencing revealed an *APC* mutation and a *TERT* promoter mutation in both tumor parts with the former being associated with the underlining FAP, the latter being potentially associated with an adverse outcome. In addition, the poorly differentiated component harbored mutations like *PIK3CA* and *PIK3CB* and an amplification in *FGFR1*, supporting evidence in conjunction with immunohistochemistry and conventional morphology of squamous cell morphology. The cell of origin of this component and whether this reflects an ultimobranchial pouch-related cellular component remain unclear. The patient was treated with total thyroidectomy and radioiodine ablation. On 2-year follow-up, she was disease-free with no clinical, biochemical, or structural evidence identified on risk-appropriate follow-up studies [28–30].

## Data Availability

Not applicable.
